# Identification of Spin Bowling Deliveries with an Advanced Smart Cricket Ball

**DOI:** 10.3390/s24227106

**Published:** 2024-11-05

**Authors:** Franz Konstantin Fuss, Batdelger Doljin, René E. D. Ferdinands

**Affiliations:** 1 Chair of Biomechanics, Faculty of Engineering Science, University of Bayreuth, D-95447 Bayreuth, Germany; 2 Division of Biomechatronics, Fraunhofer Institute for Manufacturing Engineering and Automation IPA, D-95447 Bayreuth, Germany; 3 Smart Products Engineering Program, Swinburne University, Melbourne, VIC 3000, Australia; 4 Discipline of Exercise and Sport Science, Sydney School of Health Sciences, Faculty of Medicine and Health, The University of Sydney, Sydney, NSW 2141, Australia; edouard.ferdinands@sydney.edu.au

**Keywords:** smart cricket ball, spin bowling, classification, gyroscope, rotation matrix, spin axis, benchmarking, finger spin, wrist spin, profiling

## Abstract

The type of throw of a spin bowler can be analysed in the laboratory using a motion analysis system. However, there is still no method to determine the type of throw using other means and less effort. To solve this problem, we revised the traditional classification of spin bowling throws and analysed whether spin bowling throws are separate entities or continuous concepts. We used an advanced smart cricket ball with high-speed gyroscopes to record the bowling actions and mathematically transformed the spin axis from the ball coordinate system (BCS) to the global coordinate system (GCS). We developed a visualisation method to map spin bowling throws from the yaw and pitch angles of the ball’s spin axis in the GCS. We compared the data from the smart ball with the data from the motion analysis system and profiled seven spin bowlers using the new method. The results of this study have shown that spin bowling throws are continuous concepts and that all differences between the two spin axis measurement methods were within 95% limits of agreement. The Smart Ball is sufficiently accurate to measure the direction of the ball’s spin axis in the GCS and is therefore well suited for profiling spin bowlers. Hybrid deliveries between sidespin, top/backspin, and swerve maximise the deviations of the ball in flight from the straight flight path in all three planes of the GCS. Hybrid throws between sidespin, top/backspin, and spin maximise the ball’s deviation from the straight trajectory in all three planes of the GCS.

## 1. Introduction

Cricket is a sport that features a direct battle between batters and bowlers. Bowlers perform an analogous role to the pitchers in baseball, seeking to dismiss the batters while conceding as few runs as possible. They fall into two broad categories: fast bowlers, who deliver the ball at high speeds to reduce the batter’s reaction time [1,2], and spin bowlers, who apply spin torque with their fingers, causing the ball to deviate off the pitch through friction [3]. Unlike fast bowling, spin bowling poses a more intricate challenge, forcing the batter to anticipate changes in the ball’s vertical and horizontal trajectories both in flight and after bouncing off the pitch [4,5]. This creates a unique battle of skill and strategy between batter and bowler, as the batter must adjust both body and bat to respond to the subtle variations in flight and movement.

Spin bowlers use a variety of deliveries to exploit match conditions, pitch characteristics, and batter weaknesses, making spin bowling a highly strategic and adaptable aspect of cricket. Different types of deliveries, such as wrist spin and finger spin, are bowled not just for variety but to keep the batter guessing and maximise the bowler’s effectiveness. Wrist spin, for instance, can generate more spin and varied trajectories, which are particularly effective on turning pitches, whereas finger spin offers greater control and consistency, allowing bowlers to maintain pressure over long periods. These tactical variations enable spin bowlers to adapt their approach based on the state of the game, ensuring that they remain unpredictable. Additionally, the implementation of specific techniques to change the pitch angle of spin to induce swerve via the Magnus force and the invention of advanced variations like the googly, doosra, or carrom ball add further nuance to a bowler’s repertoire, allowing them to unsettle even the most experienced batters by changing spin, bounce, and flight.

**Figure 1:**
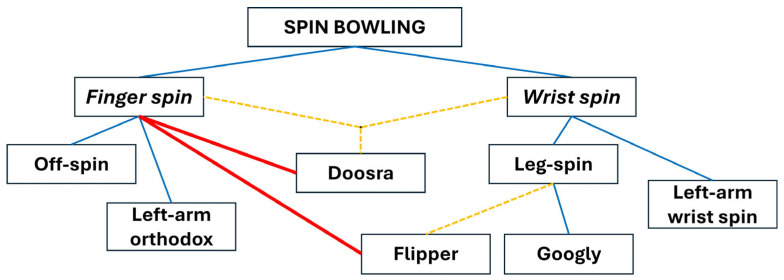
Traditional spin bowling classification tree; yellow dashed lines: incorrect classification by Woolmer et al. [6] and Baker [8]; red lines: corrections made by Fuss et al. [10].

Historically, spin bowling was categorised into wrist spin and finger spin [4,6] (Figure 1) without the benefit of a formal taxonomic framework. This classification, unlike the structured systems found in fields such as zoology [7], emerged from tradition rather than a detailed analysis of the biomechanics involved. Though still widely used, the terms wrist spin and finger spin are oversimplifications that do not account for the complex contributions of the forearm, wrist, and fingers in generating spin. A more accurate taxonomy would consider these factors. Yet, the basic labels remain useful for distinguishing between the two main methods of spinning the ball, based on the deviation they create relative to the off- and leg-sides of the cricket field [3,6]. The evolution of different spin techniques has been driven by the tactical needs of the game, making spin bowling an essential element of cricket’s strategic complexity.

A foundational classification taxonomy for spin bowling deliveries has been proposed in the form of a diagrammatic tree, illustrating the relationships between various types of spin deliveries [3,6,8] (Figure 1). This taxonomy lists the googly and the flipper as variations within the wrist-spin category and considers the doosra as a hybrid form of finger spin and wrist spin. However, a subsequent analysis of these deliveries using the world’s first smart cricket ball [9] revealed that the doosra and the flipper are in fact finger spin deliveries [10]. This conclusion was drawn from analysing the directions of the angular velocity vectors of these two deliveries with respect to the bowler’s hand, highlighting the need for a formal taxonomy of spin bowling based on quantifiable kinematic and kinetic variables. A classification system grounded in these scientific metrics, such as spin rate, axis of rotation, and torque, provides a more objective and precise means of categorising spin deliveries, moving beyond subjective interpretations. Such an approach not only deepens the understanding of current techniques but also enables the discovery and systematic classification of new variations, advancing spin bowling as a more scientifically informed discipline [11,12].

To develop a more precise and scientifically grounded classification of spin deliveries, it is crucial to accurately measure and analyse the angular velocity vectors of the ball. The methods used to determine these vectors are central to refining spin bowling taxonomy. To this end, the standard approach involves employing a motion analysis system with a ball marked with at least three indicators, a technique applied in various sports. This method has been utilised for tennis balls [13] and cricket balls [4,14] and similarly in baseball, where high-speed cameras and markers have been used to assess angular velocity vectors [15,16,17].

Another method was adopted by McGinnis et al. [18] and McGinnis and Perkins [19] by attempting to measure the angular velocity vector in the global coordinate system (GCS) using a baseball instrumented with an IMU (inertial measurement unit). They calculated the translational velocity by integrating the acceleration at the centre of mass of the ball and correcting the drift error via polynomial approximations. The drift error was calculated from motion analysis data obtained from the ball “*coated in reflective tape*”. McGinnis and Perkins [19] reported the residual error of the translational velocity (corrected velocity compared to the velocity obtained from the motion analysis data) but failed to report the error of the orientations of the translational and angular velocity vectors in the GCS.

Despite these advancements, challenges remain in accurately profiling spin bowlers in cricket. To date, there is no convincing and accurate method available for profiling spin bowlers that automatically identifies the type of delivery. Current laboratory-based motion analysis systems, which use marker positions on the ball, do not fully eliminate measurement errors. The inability of traditional marker systems to maintain a rigid body and the limitations of temporal filters, such as low-pass Butterworth filters, underscore the need for more reliable methods. As noted by Fuss et al. [20], these persistent issues highlight the ongoing difficulty in automatically and accurately identifying spin delivery types in cricket.

To address these limitations, smart cricket balls equipped with embedded gyroscopes have been introduced to directly measure angular velocity around the ball’s three axes. These smart balls provide angular velocity data within the ball coordinate system (BCS) but do not directly translate this information to the global coordinate system (GCS). Due to the high spin rates generated by spin bowlers, often exceeding 40 revolutions per second (rps) [21], smart cricket balls must be equipped with high-speed gyroscopes capable of measuring up to 55 rps [9]. However, commercially available smart cricket balls, such as the Kookaburra ball [22], are unable to determine the exact position of the spin axis because they lack high-speed gyroscopes with the necessary specifications. Instead, these balls estimate spin rate based on the frequency of magnetometer signals during flight [23], providing only a single data point, compared to the 815 data points per second produced by more advanced smart cricket balls [9,10]. While the Kookaburra smart ball was designed “*to offer a fun, engaging and educational platform that lets players, coaches, and fans engage with the sport on a whole new level*” [24], and is marketed as a tool that “*measures your bowling performance*” [25], it is not capable of accurately detecting or classifying the various types of spin deliveries bowled by players.

Professional spin bowlers can benefit significantly from an analysis of spin bowling deliveries and performance using data from an advanced smart cricket ball [9,10]. Hence, this study aimed to (1) establish a new classification system for spin bowling deliveries; (2) evaluate whether the deliveries are best treated as nominal or continuous entities; (3) transform the angular velocity (**ω**) vector from the ball coordinate system (BCS) to the global coordinate system (GCS); (4) validate the accuracy of the ball-derived orientations of the **ω**-vector in the GCS by comparing them with motion analysis data; and (5) propose a new method for assessing delivery performance to enhance the profiling of spin bowlers.

## 2. Materials and Methods

### 2.1. The World’s First Smart Cricket Ball

The advanced smart cricket ball used in this study, developed by Fuss et al. in late 2011 [9], is equipped with three high-speed gyroscopes capable of measuring angular velocity up to 20,000°/s (55 rps) [9,10]. The ball records the data at 815 Hz and transmits the data wirelessly to a laptop or smartphone. The ball is inductively charged. The angular velocity data are filtered using a third-order low-pass Butterworth filter at a cutoff frequency of 33 Hz [10]. The ball is fully balanced, and the electronics unit including gyros, a battery, and a Bluetooth transmitter has a diameter of 30 mm. The ball is encased in a conventional cricket ball leather cover. The ball is carefully calibrated [9], as the angular velocity must be measured precisely, a required prerequisite in the transformation of the **ω**-vector from BCS to GCS. The method of aligning the sensor coordinate system (SCS) to the BCS (*z*-axis perpendicular to the plane of the seam) is explained in Fuss et al. [10]. The ball is capable of measuring and calculating 5 physical performance parameters (angular velocity, resultant torque, spin torque, angular acceleration, and power) and 5 skill performance parameters (precession, normalised precession, precession torque, efficiency, and frequency) which are described in detail in Fuss et al. [10].

### 2.2. Classification of Spin Bowling Deliveries Based on the Direction of the Angular Velocity Vector in the Global Coordinate System

**Figure 2:**
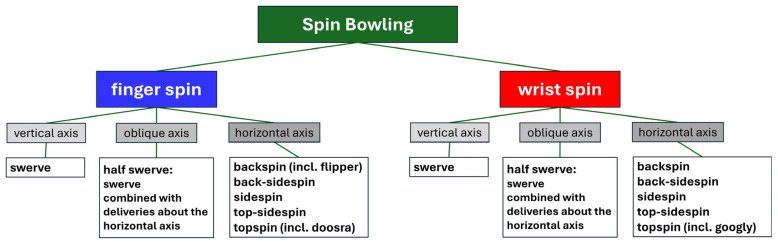
New spin bowling classification tree proposed in this study.

In spin bowling, it is essential to differentiate between two fundamentally distinct delivery techniques [10] (Figure 2): finger spin (FS), characterised by ulnar deviation of the fingers at the metacarpophalangeal joints, and wrist spin (WS), involving radial deviation of the fingers. Each of these techniques is divided into backspin (BS), back-sidespin (BSS), sidespin (SS), top-sidespin (TSS), topspin (TS) [10], and swerve (SW). In BS and TS deliveries with a forward-directed flightpath in the GCS, the corresponding **ω**-vectors point to the right and to the left, respectively, regardless of handedness and FS/WS. For all other sub-deliveries, the orientation of the **ω**-vector depends on the combinations of handedness and FS/WS deliveries. For SS deliveries, the **ω**-vector is sagittal, whereas for SW it is vertical.

It is important to note that this classification differs from baseball’s. Although both sports share the same orientation of the **ω**-vector for topspin (TS) and backspin (BS) (as outlined in Table 1 of Whiteside et al. [17]), the terminology varies. In baseball, sidespin matches what cricket refers to as swerve (vertical **ω**-vector), while rifle spin (gyroball) in baseball corresponds to sidespin in cricket (sagittal **ω**-vector).

**Figure 3:**
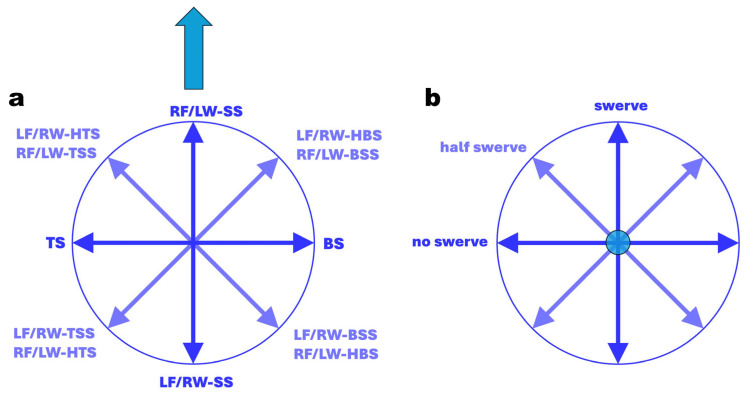
Explanation of separate, so-called “*perfect*” entities of spin bowling deliveries; (**a**): global coordinate system (GCS), top view; the thick blue arrow indicates the direction flight path of the ball; the 8 thin blue arrows represent the yaw angle of the angular velocity vector in plan view of the GCS (e.g., for RF/LW-SS, the yaw angle s 0°; for TS, the yaw angle is +90°); (**b**): GCS in front (batsman’s) view; the blue circle indicates the flight path vector in front view; the 8 thin blue arrows represent the pitch angle of the angular velocity vector in front view of the GCS (e.g., for ‘no swerve’, the pitch angle s 0°; for swerve, the yaw angle is ±90°). LF: lefthanded finger spin; RF = righthanded finger spin; LW: lefthanded wrist spin; RW = righthanded wrist spin; SS = sidespin; TSS = top-sidespin; TS = topspin; HTS = hyper-topspin; BSS = back-sidespin; BS = backspin; HBS = hyper-backspin.

**Figure 4:**
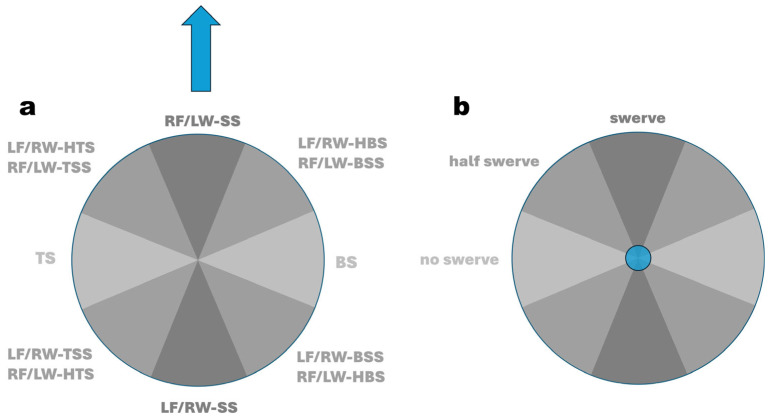
Explanation of continuous, adjacent entities of spin bowling deliveries; (**a**): global coordinate system, top view (the blue arrow indicates the direction flight path of the ball); (**b**): global coordinate system front (batsman’s) view (the blue circle indicates the flight path vector in front view); LF: lefthanded finger spin; RF = righthanded finger spin; LW: lefthanded wrist spin; RW = righthanded wrist spin; SS = sidespin; TSS = top-sidespin; TS = topspin; HTS = hyper-topspin; BSS = back-sidespin; BS = backspin; HBS = hyper-backspin.

In analogy to a wind rose, the subcategories of spin deliveries, where the **ω**-vector lies in the horizontal plane (azimuth or yaw angle), are distributed in 45° increments around a circle (Figure 3). These deliveries can be viewed as distinct or “perfect” entities, as classified by Whiteside et al. [17], or, as proposed in this study, as continuous, adjacent entities (Figure 4) with a bandwidth of ±22.5°. This approach removes the need for the term “perfect delivery”.

Special deliveries such as the flipper, googly, and doosra also fit into this classification. However, alternative functional categories proved to be more accurate in describing the mechanics of the deliveries. For instance, the flipper, contrary to Woolmer et al. [6], was classified as a special type of finger-spin backspin delivery [10], not a wrist spin (Figure 1). In addition, the googly was a specific wrist-spin topspin delivery, while the doosra is a unique finger-spin topspin variation.

Similarly, sub-deliveries with the **ω**-vector in the vertical plane (elevation or pitch angle) are also organised in 45° increments around a circle (Figure 3 and Figure 4). Deliveries without swerve fall within a pitch angle range of −22.5° to +22.5°, while swerve deliveries are characterised by pitch angles between |67.5|° and |90|°. Hence, for any delivery falling between |22.5|° and |67.5|°, we recommend using the term “half-swerve”.

### 2.3. Decision Making for Classifying Spin Bowling Deliveries as Separate Entities or Continuous and Adjacent Entities

Based on the two options shown in Figure 3 and Figure 4, the following questions arise:

(1) Should delivery identification data be analysed as nominal data (e.g., a number assigned to each delivery) or as continuous data (using the yaw and pitch angles of the **ω**-vector in degrees)?
(2) Is the concept of, e.g., a *perfect* backspin delivery, defined by the angular velocity vector being exactly at −90°, justifiable? Specifically, does this mean that deviations from −90° within a window of −90° ± 22.5° result in increasingly *imperfect* backspin?
(3) Is it practical to differentiate between *perfect* and *imperfect* executions of deliveries, or should we establish a clear boundary between distinct delivery types? For example, should a yaw angle of +22.5° be used to distinguish between deliveries with yaw angles of +22.49° and +22.51°, treating them as entirely different deliveries?

Setting aside the philosophical considerations of these questions, the technical challenge in distinguishing between a classification based on nominal versus continuous data lies in finding evidence that each delivery is executed with a distinct dynamic technique. While it is indisputable that the fundamental techniques of finger spin (FS) and wrist spin (WS) differ visually during the release phase, the orientation of the hand in the global coordinate system (GCS) ultimately determines the type of delivery. Thus, the entire bowling action, from the ball’s position under the bowler’s chin during the windup to its release, must be thoroughly analysed for dynamic variations. To achieve this, we tracked the movement of the **ω**-vector, or spin axis, across the ball’s surface within the ball coordinate system (BCS). The rate of this movement, measured in radians per second (rad/s), is defined as the precession parameter *p* [10]:

(1) $$p=\frac{T\;sin\theta }{\omega \;I}$$

where *T* denotes the total torque imparted on the ball, *θ* is the angle between the **T**- and **ω**-vectors, and *I* is the moment of inertia of the ball.

**Figure 5:**
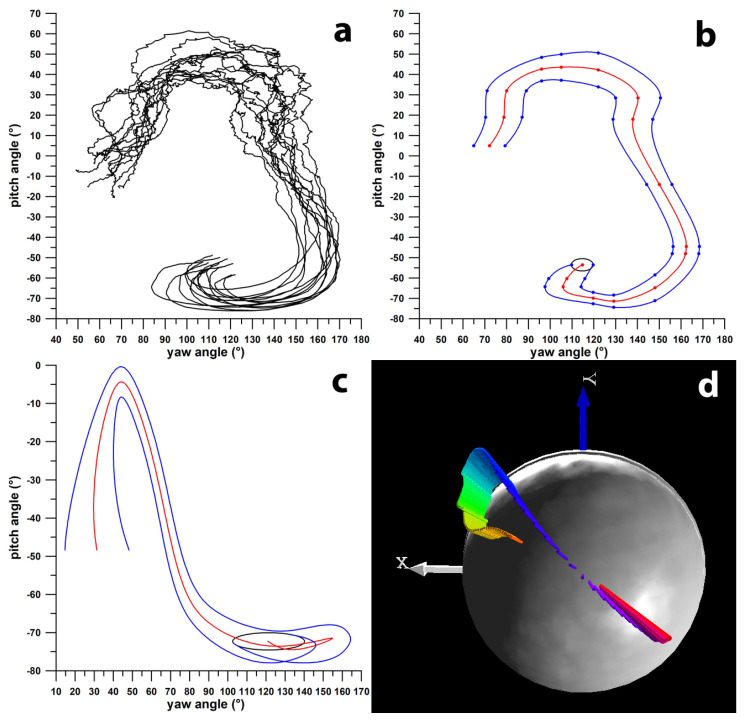
Path of the angular velocity **ω**-vector on the surface of the ball; (**a**) pitch vs. yaw angles of the **ω**-vector for finger-spin sidespin deliveries; (**b**) average path (red) ± 1 standard deviation (blue) of the date shown in (**a**); (**c**,**d**) comparison of the 2D path (yaw vs. pitch angles) of the **ω**-vector for a flipper delivery (**c**) and the 3D **ω**-vector representation for the same flipper delivery (**d**) where the time is colour-coded (**ω**-vector diagram starts with red/orange vectors and ends with purple/magenta vectors at the release of the ball); (**c**) shows the lateral distortion of the **ω**-vector path at extreme pitch angles; the black ellipse in (**b**,**c**) marks the release point.

To visualise the **ω**-vector’s position, we plotted its x, y, and z coordinates as x and y points on an equirectangular projection, using the yaw angle as longitude and the pitch angle as latitude. This projection is also referred to as a plate carrée map, where the standard parallel aligns with the equator. It is important to note that, in the plate carrée projection of the GCS, distances along the longitude become increasingly distorted near the poles, as small circles parallel to the equator’s great circle are shown with equal circumferences in the projection. Using this projection, we calculated the positions of the **ω**-vector during the windup phase by determining the intersections of the ball’s surface and the **ω**-vector (Figure 5). The path of these intersection points was averaged across at least six delivery records (one over) of the same delivery type, bowled by an experienced former first-class cricketer. The averaged path curves were investigated for similarities and differences.

### 2.4. 3D and 2D Classifications of Spin Bowling Deliveries in the Global Coordinate System

**Figure 6:**
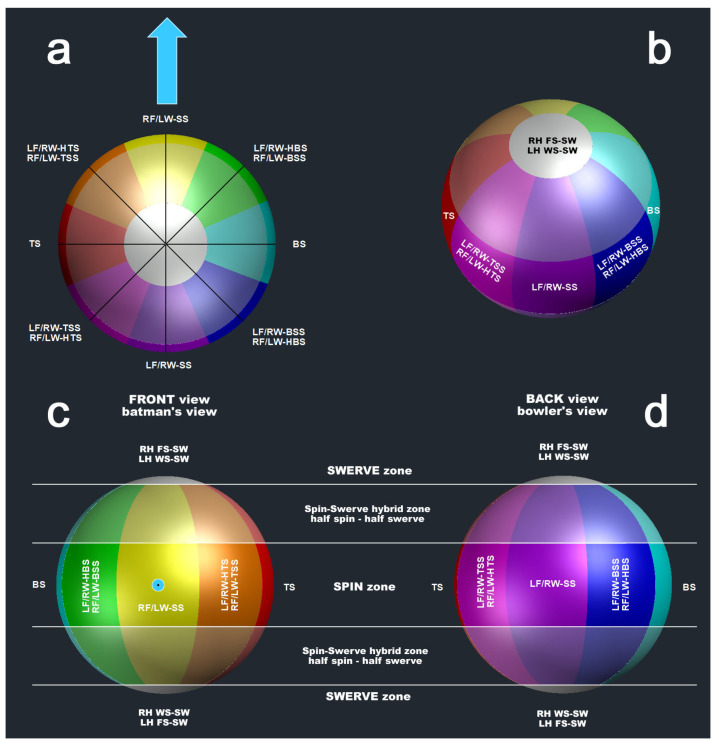
3D visualisation of the ball relative to the global coordination system (GCS); (**a**) top view; (**b**) tilted view; (**c**) front view (batsman’s view); (**d**) back view (bowler’s view); the blue arrow (**a**) and the blue dot (**c**) represent the flight path vector of the ball; the surface of the ball is divided into 8 colour-coded spherical wedges and 5 spherical segments, all of them having an included angle of 45°; LF: lefthanded finger spin; RF = righthanded finger spin; LW: lefthanded wrist spin; RW = righthanded wrist spin; SS = sidespin; TSS = top-sidespin; TS = topspin; HTS = hyper-topspin; BSS = back-sidespin; BS = backspin; HBS = hyper-backspin; SW = swerve; note that the coloured areas projected on the spinning ball are stationary in the GCS; if the angular velocity vector (stationary in both BCS and GCS) intersects the ball’s surface in the yellow area, the delivery is a sidespin (for righthanded finger spinners and lefthanded wrist spinners).

To anticipate the results of the previous sub-study, the applicability of the classification shown in Figure 4 was demonstrated. Therefore, we initially solved the identification of the deliveries in the GCS in 3D by assigning regions on the surface of a ball flying forward in the GCS (towards the positive *x*-axis) to the corresponding and expected positions of the **ω**-vector for each delivery (Figure 6).

The region assigned to each throw is the surface of a spherical wedge (Figure 6) in the plan view. There are a total of 8 wedges with an included yaw angle of 45°. These wedges are divided into 5 spherical segments in the side view. Each of these sectors has an included pitch angle of 45°, which corresponds to the no-swerve (or spin), half-swerve, and swerve zones (Figure 6).

**Figure 7:**
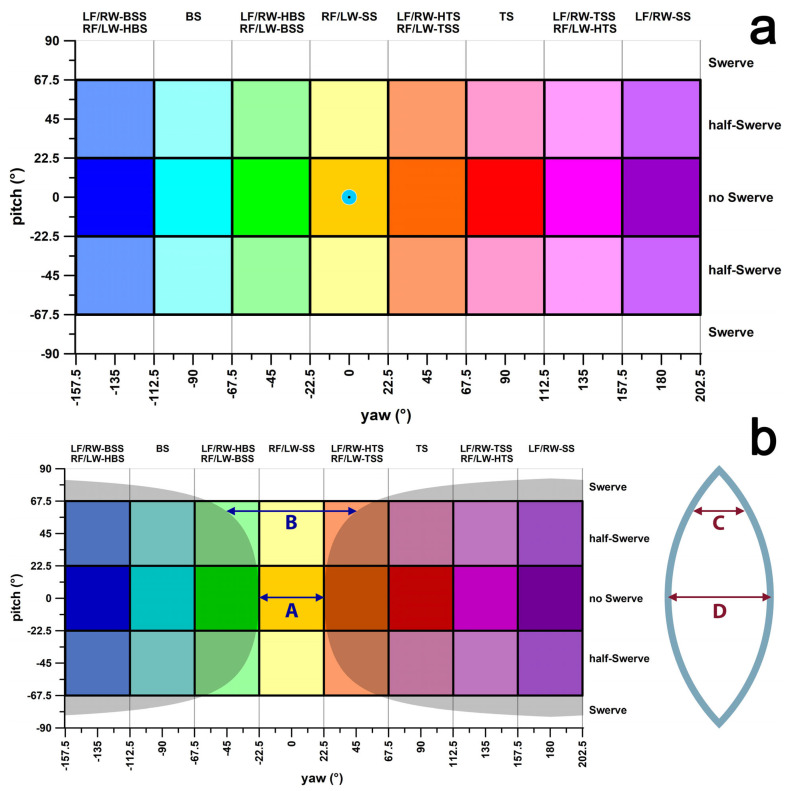
Plate-carrée view of Figure 6, showing the pitch vs. yaw angle diagram (**a**) of the ball’s angular velocity vector in the global coordinates system (GCS); blue circle: ball’s flight path direction vector in the GCS; the colour code is the same as in Figure 6; (**b**) illustrates the distortion of plate carrée projection, distances A and B are identical in Figure 6 but different in (**b**); distances A and D are identical; distance D is twice distance C, but the included angle of C and D are identical because D and C are on the surface of a spherical wedge; the grey zone blackens the area outside the distance of A and equivalently long distances in 3D (Figure 6) such as distance B; LF: lefthanded finger spin; RF = righthanded finger spin; LW: lefthanded wrist spin; RW = righthanded wrist spin; SS = sidespin; TSS = top-sidespin; TS = topspin; HTS = hyper-topspin; BSS = back-sidespin; BS = backspin; HBS = hyper-backspin.

While 3D images provide intuitive visualisations, they are impractical for comprehensively mapping all sides of the ball, as multiple views are required. To address this, we transformed the surface of the 3D ball into a 2D plate carrée map of the GCS (Figure 7), converting the spherical surface into a cylindrical surface. In this map, the poles of the sphere, originally represented as two 0D points, are expanded into 2D circles with radii equal to that of the sphere. The plate carrée map (equirectangular projection) plots latitude (pitch angle, *θ*) against longitude (yaw angle, *ψ*, in degrees; Figure 7a). It should be noted that circles positioned along the equator (latitude or pitch angle of 0°) become increasingly distorted into ellipses at pitch angles greater than 0°. This distortion factor, δ, corresponds to the major diameter of these ellipses, commonly referred to as Tissot ellipses [26].

If the length of the equator of a spherical wedge is 1 (arbitrary units, A and D in Figure 7b), then the sphere’s circumference (great circle) is 8. Hence, the radius *R* of the sphere is calculated as 8/(2π) = 1.2732. As the pitch angle increases, the great circle becomes a small circle (parallel to the equatorial plane), with smaller radius and circumference. Likewise, the arc length *L* of the small circle on one spherical wedge becomes shorter (C in Figure 7b). The arc length *L* = cos*θ*, since cos(0) = 1 when *θ* = 0 (*L* on great circle). The distortion of *L* on the plate carrée map has a distortion factor δ of δ = cos^−1^*θ* = sec*θ*. At *θ* = 60°, *L* = 0.5 (C in Figure 7b) and δ = 2 (B in Figure 7b). If the positions of a bowler’s **ω**-vectors (spin axes) in the yaw direction scatter at an average pitch *θ* angle smaller or greater than zero, then the standard deviation of this scatter must be corrected by the distortion factor δ.

### 2.5. Transformation Procedure for Rotating the **ω**-Vector from the BCS into the GCS

When using the unfiltered raw data from the three gyro sensors, it is necessary to establish the null-offset of the zero-velocity point by holding the ball in a static position for a few seconds. For instance, when employing a microcontroller with a 10-bit encoder, the null-offset should register at 512 ASCII, with positive or negative angular velocities indicated by values above or below this threshold, respectively. Minor deviations from this datum are common and simple to calculate by averaging the static ASCII readings from each sensor. Once the x, y, and z offsets were calculated, they were subtracted from the corresponding ASCII datasets. The corrected ASCII data were then converted to angular velocity values based on the calibration results from Fuss et al. [9].

The coordinates system of the GCS was defined as follows: x—forward in the direction of the ball’s flight; y—to the left; z—up. The GCS maintains a constant coordinate system at *Xx* = 1, *Yy* = 1, and *Zz* = 1, whereas the BCS coordinate system is only initially at *Xx* = 1, *Yy* = 1, and *Zz* = 1 via its initial alignment with the GCS.

From the first instance when *ω_x_* or *ω_y_* or *ω_z_* deviates from the zero line, the BCS is continuously rotated by the amount of d*θ* (the instantaneous rotation angle) about the instantaneous **ω**-vector, which is the instantaneous *ω* times d*t*, where dt is the reciprocal of the data sampling frequency (815 Hz). A single rotation about the **ω**-vector in space requires five rotation matrices **R**:

(2) $${BCS}_{i+1}={R_{Z}}^{-1}{R_{Y}}^{-1}R_{X}R_{Y}R_{Z}{BCS}_{i}$$

where **BCS***_i_* denotes the following matrix:

(3) $$BCS=\left[\begin{array}{ccc} Xx & Xy & Xz\\ Yx & Yy & Yz\\ Zx & Zy & Zz \end{array}\right]$$

i.e., the *X*, *Y*, and *Z* axes of the BCS as vectors in the GCS at times *i* and *I* + 1, where *t*_(*i*+1)_—*t_i_* = d*t*; and where:

- **R_Z_** is the rotation matrix for a BCS-rotation about the *Z*-axis by +*α*, equivalent to rotation of the **ω**-vector into the XZ-plane:

(4) $$R_{Z}=\left[\begin{array}{ccc} cos\alpha & sin\alpha & 0\\ -sin\alpha & cos\alpha & 0\\ 0 & 0 & 1 \end{array}\right]$$

- **R_Y_** is the rotation matrix for a BCS-rotation about the *Y*-axis by +*β*, equivalent to the rotation of the **ω**-vector into the *X*-axis:

(5) $$R_{Y}=\left[\begin{array}{ccc} cos\beta & 0 & sin\beta \\ 0 & 1 & 0\\ -sin\beta & 0 & cos\beta \end{array}\right]$$

- **R_X_** is the rotation matrix for a BCS-rotation about the *X*-axis by +d*θ*:

(6) $$R_{X}=\left[\begin{array}{ccc} 1 & 0 & 0\\ 0 & cos\left(d\theta \right) & sin\left(d\theta \right)\\ 0 & -sin\left(d\theta \right) & cos\left(d\theta \right) \end{array}\right]$$

where the angles *α* and *β* are the yaw and pitch angles of the **ω**-vector, calculated as follows:

(7) $$\alpha =ATAN2\left(\omega_{y},\omega_{x}\right)$$

(8) $$\beta =ATAN2\left(\omega_{z},\sqrt{{\omega_{x}}^{2}+{\omega_{y}}^{2}}\right)$$

Then, after completing each rotation step from *t_i_* to *t*_(*i*+1)_, the BCS is rotated back into the GCS along with the original **ω***_i_*-vector, aligning the **ω***_i_*-vector with the GCS. Since the matrix of coordinate system axes of the GCS is an identity matrix, the instantaneous rotation matrix required to rotate a vector aligned with the BCS at *t_i_* into the GCS is the rotation matrix **BCS** from Equation (3).

Therefore, when the vector **ω***_BCSi_* is rotated into the GCS, the *new* orientation of the **ω**-vector (**ω***_GCSi_*) is calculated as follows:

(9) $$\omega_{GCSi}={BCS}_{i}\;\;\omega_{BCSi}$$

If the positions of the **ω**-vectors are calculated in the GCS, their yaw and pitch angles are constant at the beginning of the flight phase immediately after the release and are used to identify the type of delivery according to Figure 6 and Figure 7.

**Figure 8:**
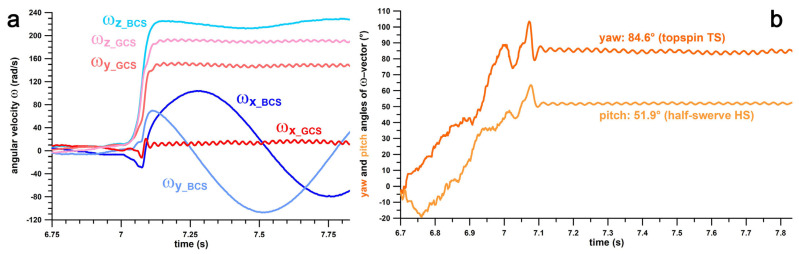
(**a**) Angular velocity ω vs. time; subscripts xyz correspond the x-, y-, and z-components of ω; BCS: ball coordinate system; GCS: global coordinate system; (**b**) pitch and yaw angles of the **ω**-vector in the global coordinate system vs. time; this diagram identifies the type of delivery.

Figure 8 shows the **ω**-components of a topspin (TS) delivery in the BCS and the GCS, as well as the yaw and pitch angles of the **ω**-vector in the GCS.

### 2.6. Benchmarking of the Smart Ball Delivery ID Data Against Those of a Motion Capture System

Since the marker data of a motion capture and analysis system (mocap) are primarily captured in the GCS, it is reasonable to expect that mocap data would be more accurate than smart ball data captured in the BCS. However, as we compare the orientation of the **ω**-vector in the GCS, both methods present advantages and disadvantages:

- Smart ball: while the angular velocity (*ω*) is measured accurately in the BCS, the process described in Equations (2)–(9) is susceptible to errors due to signal noise, filtering distortions, and insufficient sampling frequency;
- Mocap system: although the marker positions are measured precisely in the GCS, determining the exact orientation of the helical axis around which an object rotates in space requires a marker triad with rigid body properties. This means that each of the three sides of the marker triad must remain rigid. To address this limitation, we applied a novel spatial filtering method to force-fit a rigid triangle with average side lengths to the reflective marker triads attached to the cricket ball. The detailed methodology, including the calculation of the helical axis vector (which corresponds to the **ω**-vector), is described comprehensively in Fuss et al. [20].

For benchmarking we used a Cortex system (Motion Analysis Corporation, Rohnert Park, CA, USA) that measured the marker data at 200 Hz. These data were processed using the method of Fuss et al. [20] to determine the orientation of the two velocity vectors, translational velocity (**v**-vector) and angular velocity (helical axis, **ω**-vector). The **v**-vector projected onto the horizontal plane was defined as the *X*-axis of the GCS. The angle included between **v**- and **ω**-vectors, both projected onto the horizontal plane, defines the yaw angle of the **ω**-vector, which is required to identify the delivery in the horizontal plane (Figure 4 and Figure 7).

The smart ball was bowled by the same experienced bowler and former first-class cricketer mentioned above. Comparable to the method used by McGinnis et al. [18] and McGinnis and Perkins [19], the bowler picked up the ball from a tee, carefully aligning the BCS with the GCS. For this purpose, a marker was placed on the pitch anticipating the intended point of impact. In technical terms, the bowler picked up the ball and aligned the hand with the BCS (index finger at +X, and +Z pointing out of/into the palm for left/righthanded bowlers [10]). This convention served to distinguish between FS (negative *ω_z_* in flight) and WS (positive *ω_z_*). The participant then bowled the ball 31 times, covering *all* deliveries shown in Figure 4 and Figure 7. Therefore, this study focused on a single participant, chosen for his ability to consistently bowl all types of spin deliveries at a high level, supported by his extensive experience and proven skill in high-level competitions and international coaching.

We compared the pitch and yaw angles of **ω**-vectors obtained from processing the smart ball data and the mocap data for their agreement by using Bland–Altman diagrams [27,28]. For this purpose, we calculated the average and difference of two corresponding data (yaw and pitch angles, smart ball, and mocap) and plotted the difference against the average (first BA-diagrams). For swerve deliveries, the yaw data were excluded, because for a perfect swerve with a pitch angle of 90°, the yaw angle is not defined (gimbal lock). For swerve pitch angles less than 90°, the yaw angle error can be large (smart ball vs. mocap data).

Then, the smart ball data were corrected with two different methods. Bland and Altman [27] suggested correcting the bias of the difference when the average difference was nonzero, i.e., subtracting the bias from the entire dataset. The application of this correction method resulted in the second BA-diagrams diagrams.

Additionally, we employed a second method that challenges the stance of Bland and Altman [27], who argue that regression analysis is inappropriate for assessing agreement between two datasets. Bland and Altman’s objections are based on two points:

1. Agreement between datasets is inherently connected to a strong correlation;
2. Perfect correlation does not necessarily imply agreement; for instance, if one dataset’s values are consistently double those of another, they will be perfectly correlated yet not in agreement.

While we agree with these points, we applied a linear regression function solely to correct the smart ball dataset. If the two datasets are perfectly aligned, the regression function, y = *a*x + *b*, will be y = 1x + 0, where the coefficients *a* and *b* are 1 and 0, respectively.

If the regression function of the first BA-diagrams is

y = *a* x_1_ + *b* (10)

where y denotes the yaw or pitch angles obtained from the mocap system, and x_1_ denotes the original yaw or pitch angles obtained from the smart ball, and the regression function of the corrected yaw or pitch angles shall be

y = 1 x_2_ + 0 (11)

where x_2_ denotes the adjustment of the original yaw or pitch angles x_1_; then, equating Equations (10) and (11) yields

x_2_ = *a* x_1_ + *b* (12)

We used Equation (12) for an alternative correction of the original yaw or pitch angles obtained from the smart ball. This adjustment resulted in the third BA-diagrams.

### 2.7. Application for Profiling of Spin Bowlers

We profiled seven bowlers under different testing conditions:

- Bowlers A and B each bowled the same delivery both before and after an intervention;
- Bowlers C through E each performed two distinct deliveries: their standard delivery and an additional variation;
- Bowlers F and G only performed their standard delivery.

The bowlers’ performance levels ranged between amateur and first-class cricket. Each bowler delivered the ball between 6 and 12 times.

**Figure 9:**
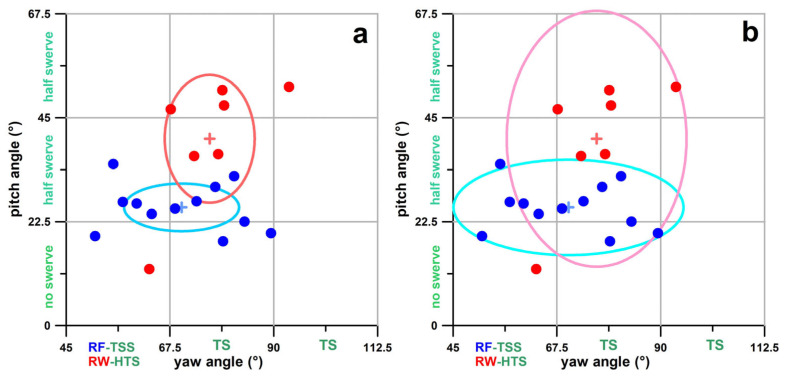
Scatter plot of pitch vs. yaw angle data of the angular velocity vectors (dots) in the global coordinate system of deliveries of a righthanded finger spinner (blue) and a righthanded wrist spinner (red); average (+) ±standard deviation (⬭); (**a**) ±one standard deviation; (**b**) ±two standard deviations; RF = righthanded finger spin; RW = righthanded wrist spin; TSS = top-sidespin; TS = topspin; HTS = hyper-topspin.

When analysing the data cluster for a single bowler, the yaw-pitch data often appear scattered (Figure 9). To effectively visualise the data distribution across all seven bowlers, it is more informative to use the average point (+) and an ellipse representing ±1 standard deviation (Figure 9a). This ellipse statistically encompasses approximately 68% of the data for yaw (horizontal axis) and pitch (vertical axis) angles. Using an ellipse with axes representing ±2 standard deviations (covering 95% of the data; Figure 9b) may lead to substantial overlap, potentially resulting in confusing comparisons between multiple bowlers.

The standard deviations in the yaw and pitch directions quantify the consistency (*C*) of a bowler delivering the same delivery. Due to the distortion of the included yaw angle of a spherical wedge on the plate carrée map, the consistency of the yaw angle of the spin axis in the GCS was corrected with the distortion factor δ (corrected *C* = original *C*/δ). The consistency of the pitch angle of the spin axis in the GCS does not need to be corrected.

## 3. Results

### 3.1. Are Bowling Deliveries Separate Entities or Continuous Concepts?

**Figure 10:**
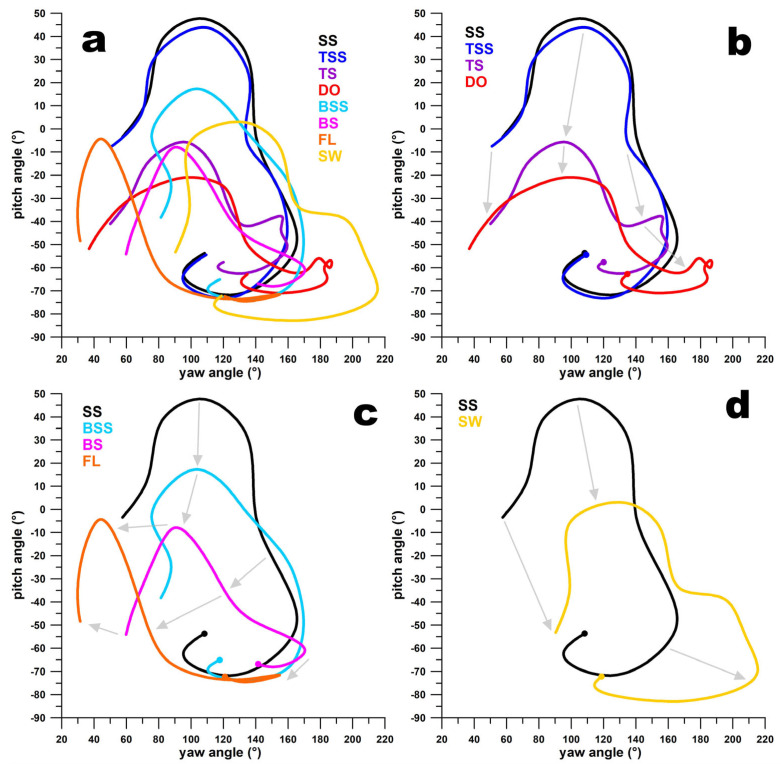
Pitch vs. yaw angles of the path of the **ω**-vector for finger spin deliveries (**a**); (**b**) transition from sidespin (SS) over top-sidespin (TSS) and topspin (TS) to doosra (DO); the dots indicate the release point; the grey arrows show that the curves flow into one another and thus represent a continuum; (**c**) transition from sidespin (SS) over back-sidespin (BSS) and backspin (BS) to flipper (FL); (**d**) transition from sidespin (SS) to swerve (SW).

**Figure 11:**
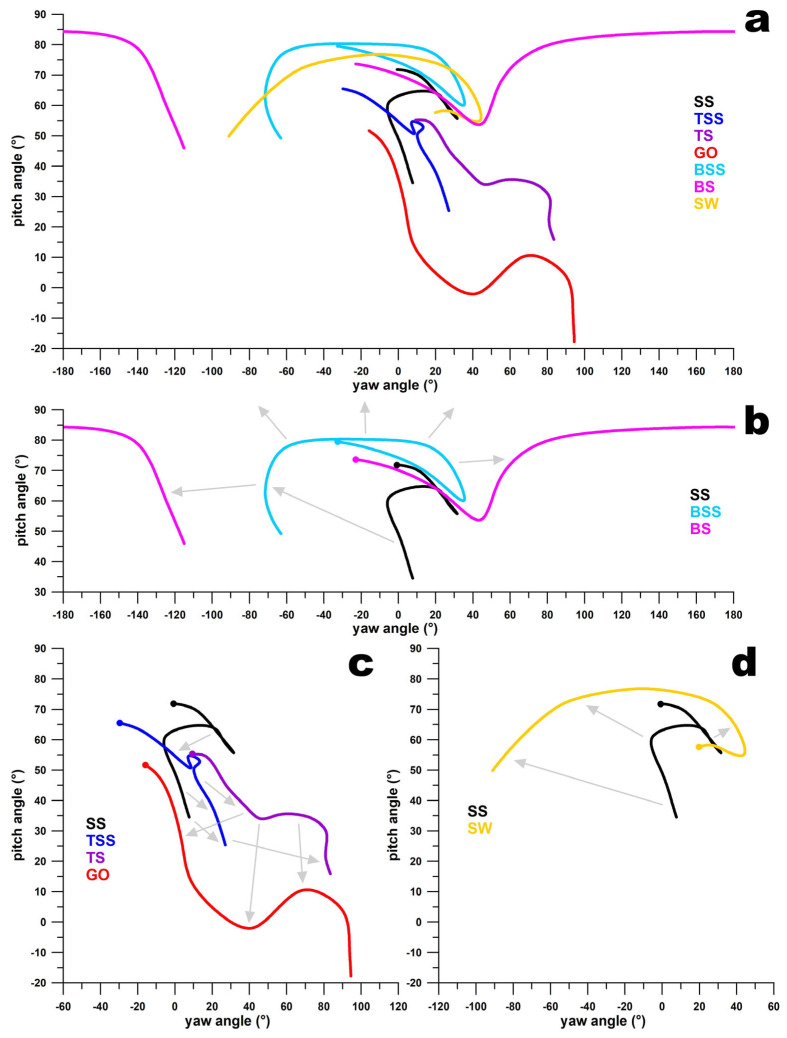
Pitch vs. yaw angles of the path of the **ω**-vector for finger-spin deliveries (**a**); (**b**) transition from sidespin (SS) over back-sidespin (BSS) to backspin (BS); the dots indicate the release point; the grey arrows show that the curves flow into one another and thus represent a continuum; the three arrows pointing upward from the blue curve (BSS) indicate that the purple curve (BS) is located on the other side of the ball’s north pole; (**c**) transition from sidespin (SS) to top-sidespin (TSS) and topspin (TS) to googly (GO); (**d**) transition from sidespin (SS) to swerve (SW).

Figure 10a–d and Figure 11a–d illustrate that the paths of the **ω**-vectors for adjacent deliveries on the smart ball’s surface are continuous. This continuity is characterised by the paths being generally translated along their coordinate axes, with only slight changes in shape. For instance, in Figure 10b, the **ω**-vector path for FS-SS and FS-TSS shows similarity, while the FS-TS path shifts towards smaller pitch angles, and the FL path shifts towards smaller yaw angles. Even though the **ω**-vector path for WS-BS initially appears discontinuous in Figure 11a,b, it actually lies on the opposite side of the ball’s north pole. It traverses through the pole to the front side of the ball, transitioning into the WS-BSS path. This path then decreases in pitch angles and increases in yaw angles, evolving into the WS-SS, WS-TSS, and WS-TS paths. The WS-TS path further shifts to smaller pitch angles and slightly larger yaw angles, ultimately transforming into the GO path.

### 3.2. Benchmarking Delivery Identification Using the Smart Ball Against the Motion Analysis Data

#### 3.2.1. Yaw Angle—Band–Altman Diagrams

**Figure 12:**
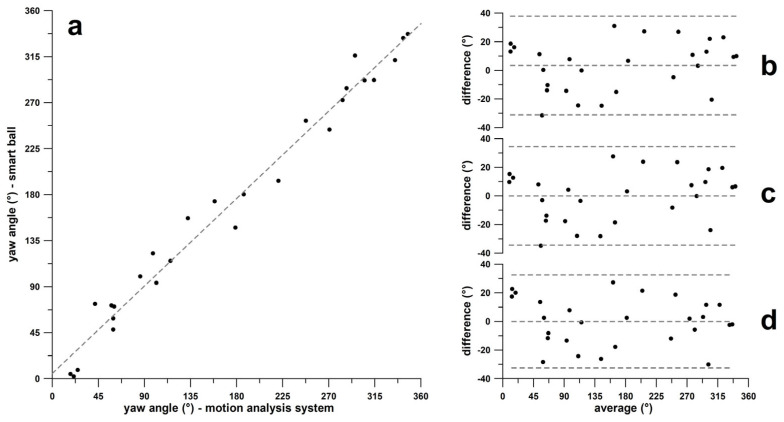
Agreement of yaw angles in the global coordinate system; (**a**) yaw angles of **ω**-vectors obtained from the smart ball vs. yaw angles derived from the motion analysis system; Bland–Altman diagrams (difference vs. average) (**b**) of the original smart ball and motion analysis data, (**c**) the data after bias correction, and (**d**) the data after correction using the linear regression function from (**a**).

Figure 12a shows the correlation plot of yaw angles obtained from the smart ball and the motion analysis system. The linear regression equation was y = 0.9505x + 5.1201 (R^2^ = 0.9773). The corresponding Bland–Altman diagrams are shown in Figure 12b–d. In Figure 12b, the bias is +3.4°, and 1.96σ corresponds to 34.42° (only one datum slightly outside the 1.96σ limit).

After adjusting for bias, the regression function was y = 0.9505x + 8.3553. In Figure 12c, the bias has been eliminated, and 1.96σ remains the same (only one datum just outside the 1.96σ limit).

After adjusting the original yaw dataset obtained from the smart ball using the regression function of Figure 12a, the new regression function was y = 1x + 0. In Figure 12d, the bias has been eliminated, and 1.96σ has decreased to 32.57° (no datum outside the 1.96σ boundary).

#### 3.2.2. Pitch Angle—Band–Altman Diagrams

**Figure 13:**
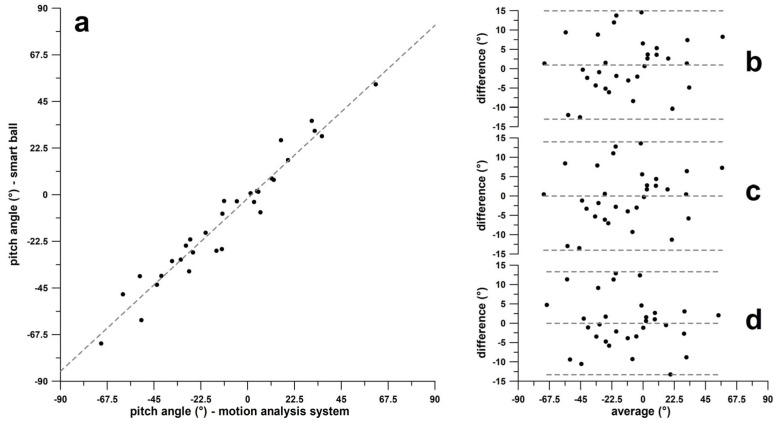
Agreement of pitch angles in the global coordinate system; (**a**) pitch angles of **ω**-vectors obtained from the smart ball vs. pitch angles derived from the motion analysis system; Bland–Altman diagrams (difference vs. average) (**b**) of the original smart ball and motion analysis data, (**c**) the data after bias correction, and (**d**) the data after correction using the linear regression function from (**a**).

Figure 13a shows the correlation plot of pitch angles obtained from the smart ball and the motion analysis system. The linear regression equation was y = 0.9275x − 1.7383 (R^2^ = 0.9466). The corresponding Bland–Altman diagrams are shown in Figure 13a–d. In Figure 13b, the bias is +0.95°, and 1.96σ corresponds to 14.0° (no datum outside the 1.96σ boundary).

After adjusting for bias, the regression function was expressed as y = 0.9275x − 0.8542. In Figure 13c, the bias has been eliminated, and 1.96σ remains unchanged. Following this adjustment, the regression function from Figure 13a was applied to the original pitch dataset obtained from the smart ball, resulting in a new regression function of y = 1x + 0. In Figure 13d, the bias has been eliminated, and 1.96σ decreased to 13.3°.

#### 3.2.3. Final Yaw–Pitch Angle Diagram

**Figure 14:**
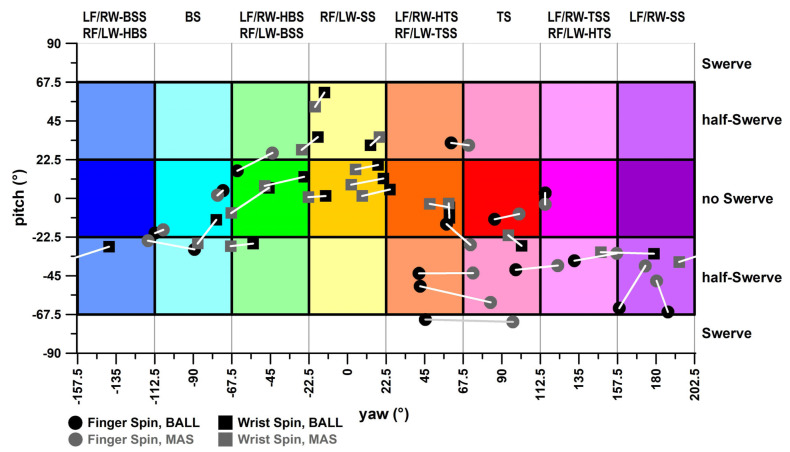
Plate carrée diagram of the deliveries and their yaw (Figure 12) vs. pitch (Figure 13) diagrams; LF: lefthanded finger spin; RF = righthanded finger spin; LW: lefthanded wrist spin; RW = righthanded wrist spin; SS = sidespin; TSS = top-sidespin; TS = topspin; HTS = hyper-topspin; BSS = back-sidespin; BS = backspin; HBS = hyper-backspin; BALL = smart ball data; MAS = motion analysis system data.

The delivery results obtained from the motion analysis system and the adjusted smart ball data (adjustment 2) are shown in Figure 14. Corresponding deliveries relate to white or grey lines (Figure 14). The equirectangular projection of the GCS shown in Figure 14 overstates the error the closer a delivery is to the ball’s pole (Figure 7b), i.e., to the upper and lower boundaries of the pitch angle at ±90°. In fact, these boundaries are not horizontal lines but rather points.

### 3.3. Examples of Delivery Identification from a Cluster Analysis

**Figure 15:**
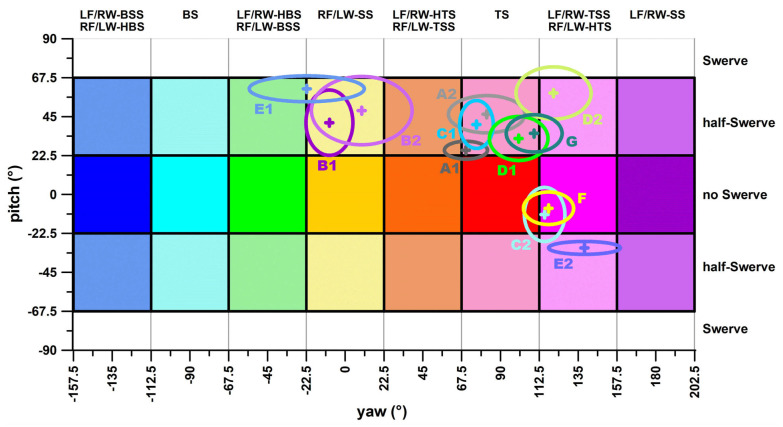
Plate carrée diagram for identification of the deliveries of 7 bowlers (A–G), bowling 12 different deliveries; 1, 2 = first and second bowling session; + = average; ⬭ = ellipse with the dimensions of ±1 standard deviation in yaw and pitch direction.

Figure 15 illustrates examples of cricket delivery identification for seven bowlers (A–G), each bowling in one or two sessions, totalling 12 deliveries. Table 1 presents the key data from Figure 15. In the yaw direction, half of the actual deliveries did not align with the intended deliveries. Among these discrepancies, three deviated by an average of one throw, while the remaining three deviated by two throws, as detailed in Table 1. Deliveries in two bowling sessions were close to the swerve zone (E1 and D2), causing a large distortion factor δ (2.06 and 1.92, respectively; Table 1). The consistency in yaw direction (standard deviation divided by δ; Table 1) ranged from 7.4° to 19.3°; and the consistency in pitch direction ranged from 3.7° to 19.7°.

## 4. Discussion

This study explored the potential of identifying spin bowling delivery types using an advanced, high-quality smart cricket ball. This task was approached by (1) fundamentally revising the traditional spin bowling classification tree and proposing a robust 3D method for improved classification, identification, and profiling; (2) rigorously benchmarking and validating the smart ball method against a motion analysis system with advanced filtering of the marker data; and (3) testing the smart ball method with seven spin bowlers and 12 delivery sessions through a profiling exercise. In this context, the data presented in Table 1, in conjunction with Figure 15, highlight the limitations of a nominal categorisation system for classifying spin bowlers. While Table 1 applies a nominal classification approach, categorising, for instance, bowler E’s delivery in session 1 as a lefthanded wrist-spinner side-spinner, Figure 15 reveals that the average yaw angle of −22.2° falls on the boundary between sidespin and back-sidespin (yaw angle of −22.5°). Although −22.2° remains within the sidespin range (>−22.5), an angle of −22.6° would already be classified as back-sidespin (<−22.5).

This issue is similarly evident for bowlers A (session 1), C (session 2), and G, suggesting that a rigid classification system with mutually exclusive categories may not adequately capture the nuances of spin bowling deliveries. Therefore, a more flexible classification approach is recommended, particularly when visualised in a classification diagram like Figure 15. In the cases of bowlers E1, A1, C2, and G, it is evident that the delivery classifications based on yaw angles are subject to variability and may be considered *imperfect*.

### 4.1. The Problem of Perfect and Imperfect Deliveries

The term *perfect* was coined by Whiteside et al. [17] for baseball pitches. From Table 1 it is evident that in 10 out of the 12 bowling sessions the bowlers executed with a half-swerve; although, none of them bowled with that intention. Is a half-swerve, a hybrid between spin and swerve, considered an imperfect delivery? Does it reflect the abilities of an inexperienced or developing bowler? The gradient classification method mentioned earlier challenges the notion of a *perfect* delivery by introducing more flexibility in how deliveries are categorised. Table 1 illustrates the differences between the intended type of delivery and the actual outcome. Distinguishing between the two suggests a deliberate choice by the bowler, but is bowling always a conscious action? A bowler might consistently deliver a particular type of ball without critically assessing its true nature, simply assuming it to be of a specific type. Moreover, these deliveries are rarely measured using advanced technology, such as biomechanical analysis or smart balls.

The discrepancy between perceived and actual movements is a well-known phenomenon in motor control and learning, as shown in the study of Shorten and Pisciotta [29]. They discovered that while runners (wear testers) self-reported foot contact as 32% heel-strike, 54% midfoot-strike, and 14% forefoot-strike, observational data from race participants revealed distributions of 94%, 4%, and 2%, respectively. Likewise, spin bowlers could adhere to the traditional classification system shown in Figure 1, likely unaware of performances that show the nuanced distinctions shown in Figure 2, Figure 3, Figure 4, Figure 6, and Figure 7. They even commonly use general terms like off-spin and leg-spin without recognising the finer distinctions, such as sidespin, top-spin, or backspin.

The notion of a *perfect* delivery becomes irrelevant in this context because a skilled bowler’s primary objective is to produce a variety of outcomes, each tailored to specific game situations. Bowlers aim to generate different spin characteristics upon rebound and manipulate varying Magnus forces to create different types of swerves. This variation is essential to deceiving the batter, as it makes each delivery unpredictable. Importantly, the selection of deliveries is often strategic, influenced by the sequence of previous balls, and designed to exploit a batter’s weaknesses. By constantly varying spin, rebound, and flight, bowlers ensure that no one delivery can be classified as *perfect* in a rigid sense, as success in the game depends on unpredictability and adaptability. Hence, Figure 15 illustrates the distribution of delivery types for spin bowlers, offering coaches valuable insight. Similar diagrams can be generated for top-level bowlers using international cricket databases (e.g., [30]), further emphasising the need for variation in delivery to achieve optimal performance and outwit the batter.

**Figure 16:**
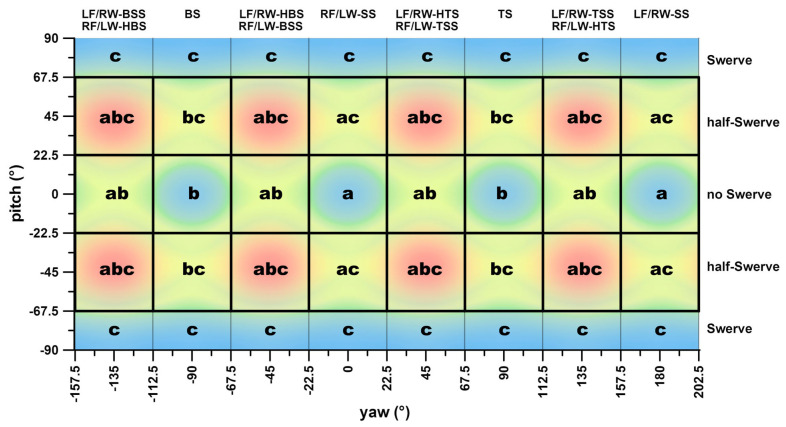
Plate carrée diagram of Figure 7a, identifying the combinations of mechanical principles for each delivery; a,b,c refer to the different mechanical principles per sub-delivery according to the text in Section 4.1 (a: sideward bounce; b: lift up/down; c: sideward flight deviation).

A highly skilled spin bowler can manipulate the application of spin torque about the three rotational axes (‘abc’ in Figure 16) to produce such variations and deceive the batsman:

- The sagittal axis (forward–backward; *x*-axis in the global coordinate system) is linked to sidespin. This spin influences the ball’s rebound direction through friction when it contacts the pitch (‘a’ in Figure 16);
- The horizontal axis (left–right; *y*-axis in the global coordinate system) is associated with top- and backspin. These types of spin impact the ball’s aerodynamics through the Magnus effect. Backspin generates an upward (positive) lift force, while topspin produces a downward (negative) lift force (‘b’ in Figure 16);
- The vertical axis (up–down; *z*-axis in the global coordinate system) relates to the swerve, which also utilises the Magnus effect. This causes the ball to curve in flight, creating a leftward side force when the angular velocity is positive (‘c’ in Figure 16).

This implies that the spin bowler can combine aerodynamic effects (up–down and left–right) with rebound kinematics (left–right) to produce a large range of variations, including hybrid deliveries. For instance, a delivery such as a half-swerve, when combined with either top-sidespin or back-sidespin, optimally utilises these mechanical factors, causing the ball to swerve laterally in flight before deviating after the bounce, increasing the complexity of predictive ball tracking by the batter. Therefore, rather than being considered an *imperfect* delivery, a half-swerve should be regarded as an *elite* delivery, even if it is executed instinctively. This further supports the need for a gradient classification system, as it accounts for the nuanced variations in deliveries that skilled bowlers use to produce different outcomes, such as bounce and flight deviations, strategically designed to deceive the batter.

### 4.2. The Gold Standard Problem

This study compares two different methods for identifying bowling deliveries. Typically, such comparisons involve evaluating a new method against a recognised gold standard. However, in this case, it is unclear which method should be considered the gold standard, as both have distinct advantages and disadvantages.

One key advantage of a motion analysis system is its ability to accurately determine the ball’s flight path based on raw marker data. If the trajectory is linear, the average flight path can be calculated. However, for curved trajectories, instead of using the straight-line distance between the release point and impact point, predictive projectile equations of motion, incorporating air resistance, could provide a more accurate model of the ball’s trajectory. This method accounts for aerodynamic effects, such as drag and lift, offering a refined prediction of the ball’s path if the measurement space in the laboratory is sufficiently large. The projected flight path on the horizontal plane corresponds to a yaw angle of 0° in the global coordinate system (GCS) (Figure 4a and Figure 6a).

However, the motion analysis system has limitations. The small side lengths of the marker triad on the ball result in higher relative noise in the marker data, which degrades the accuracy of the helical axis calculation, crucial for determining the spin axis and angular velocity vector [20]. Traditional temporal filters, such as low-pass Butterworth filters, do not address this issue as they fail to stabilise the marker data needed for accurate helical axis calculations. In fact, these filters can exacerbate the relative noise in the triad side lengths [20]. In addition, the motion analysis method is restricted to large laboratory environments with substantial measurement volumes, and the ball must be fitted with markers, further limiting its practical application.

The smart ball offers the advantage of accurately calculating the spin axis (angular velocity vector) based on raw angular velocity data that can be obtained outdoors on the playing field without the interference of markers on the ball. However, there are limitations when converting the BCS to the GCS, which impact the accuracy of this study. The gyros embedded in the smart ball are designed for high-speed measurements up to 20,000°/s (55 rps). As a result, slow movements of the ball, such as pre-delivery spins or other non-standard actions, can introduce errors. These inaccuracies affect the precision of calculating yaw and pitch angles of the spin axis. Another limitation is related to the initial alignment of the BCS with the GCS. The *z*-axis of the BCS must point upwards, while the *x*-axis should align with the ball’s flight path. Ideally, the *x*-axis could be directed towards the wicket or the impact point on the pitch, assuming accurate visualisation by a marker. However, the flight path can only be approximated, not precisely aligned.

Given that errors in calculating the direction of the angular velocity vector in the GCS are stochastic rather than systematic, it is advisable to record at least two overs (12 deliveries) to calculate the averages and standard deviations of yaw and pitch angles. This approach helps in identifying the type of delivery through cluster analysis, as illustrated in Figure 15.

An important consideration is the availability of an experienced spin bowling coach to eliminate all extraneous movements of the ball that occur before the period when the fingers begin applying spin torque. A failure to do so would lead to inaccurate calculation of the spin axis and angular velocity vector. Consequently, the first step in the experimental protocol of using an advanced smart cricket ball is to implement a standardised protocol for ball handling and delivery preparation to minimise unwanted movements. For instance, establish specific guidelines for how the ball should be held and positioned before the wind-up to ensure consistency. For example, instructing the bowlers to avoid spinning or rotating the ball unnecessarily before the actual delivery. This reduces the risk of capturing irrelevant data that could skew the spin measurements. The second step is to confirm the validity of the movement elimination protocol, by segmenting the data based on time intervals to focus on the period after the development of spin torque.

Determining which method, motion analysis or smart ball, should be considered the gold standard and is straightforward within the context of this study, since we applied a spatial filter [20] to process the marker data. This spatial filter effectively rigidifies the marker triad, leading to a more accurate calculation of the spin axis direction in the global coordinate system (GCS). While the smart ball offers the advantage of on-field profiling and direct angular velocity data, the task of aligning the ball coordinate system (BCS) with the GCS and removing extraneous movements from the dataset requires careful administration.

### 4.3. Application to Coaching

To address the increasing complexity of cricket, coaches can benefit from an understanding of the sophisticated techniques underlying the performance of elite spin bowlers, mirroring the coaching evolution seen in other sports. Many competitive sports have integrated complex, abstract techniques into training as the demand for nuanced skill sets grows. Sports like golf, baseball, and tennis each rely on intricate biomechanics and high-level motor coordination that are integral to player performance. Hence, optimising the performance of spin bowlers will require coaches to embrace structured, science-based approaches to skill acquisition [31]. For instance, coaches can employ analogical learning, which has been used successfully in other sports [32], to learn certain types of spin deliveries by relating to other familiar skills such as curving a soccer ball in an instep kick or swerving a baseball in a fast-speed pitch [32]. Cricket organisations could also invest in biomechanics-based coaching programs that include hands-on workshops with feedback tools and simulation apps that deliver real-time spin delivery data from the smart cricket ball to coaches and spin bowlers.

Special deliveries such as doosra DO, flipper FL, and googly GO, although clearly shown as separate entities in Figure 10b,c and Figure 11c, would be expected as topspin (DO, GO) and backspin (FL) in Figure 7a. They can be further distinguished from traditional top- and backspin deliveries by considering other performance parameters provided by the smart ball [10]. For example, DO is more efficient than FS-TS, GO is less efficient than WS-TS, and FL is less efficient than FS-BS. Interestingly, in relation to the doosra versus sidespin (off-spin or off-break), Woolmer et al. [6] point out that from the batsman’s view, “*the off-break delivery should show the batsman* … [the] *watch-strap*” of the bowler’s wristwatch, “*while the doosra should show him the time*”. This analogy implies that the doosra is a sidespin throw, where the ball is spun in the opposite direction to the traditional sidespin if the palm of the hand does not block the ball’s trajectory upon release. Nevertheless, it is likely that at least some doosras are hyper-topspin deliveries rather than just pure topspinners. Further research is required to fine-tune the delivery classification of doosra and its variations within the proposed classification chart (Figure 7a and Figure 15).

This study advances the profiling of spin bowlers by integrating a comprehensive range of performance parameters. Utilising the smart ball developed in late 2011 [9], which can measure five physical and five skill performance parameters [10], this research enhances the current methods of bowling delivery identification. By combining these detailed performance metrics with the new delivery identification techniques, coaches and bowlers gain access to an extensive dataset. This wealth of information can be leveraged not only for in-depth profiling but also for conducting intervention studies [10], assessing performance improvements, evaluating the execution quality of different deliveries, and documenting the variety of deliveries a bowler can master.

In contrast, the Kookaburra smart ball, which Kookaburra claims, but incorrectly, to be “*the world’s first microchipped cricket ball*” [22] (available on the market since 2020 whereas our advanced smart ball was developed in 2011 and published in 2012 [9,33]), only measures one translational velocity value and one angular velocity value after release. This information is not sufficient for the profiling of spin bowlers. Fuss et al. [10] have clearly shown that, for example, topspin deliveries are more efficient than backspin deliveries and that topspin deliveries, therefore, generate comparatively more spin than backspin deliveries. A single angular velocity value, therefore, does not show a complete picture of a spin bowler’s performance.

In contrast to this, the advanced smart ball [9,10] can be applied by coaches as an effective coaching tool to improve spin bowling performance via an augmented instructional process, outlined as follows.

The 3D visualisation described in Figure 6 has several valuable coaching applications for spin bowling:

- *Spin analysis and identification*: the colour-coded wedges and segments help coaches and players easily identify the type of spin (e.g., topspin, backspin, sidespin, or hyper variations) applied by bowlers. This can be used to analyse and fine-tune the bowler’s technique and ensure consistency in spin delivery;
- *Advanced coaching education*: biomechanists can use this visualisation to instruct coaches on how the different types of spin affect the ball’s flight path. By showing the angular velocity vector relative to the ball’s surface, coaches can help bowlers appreciate how subtle changes in grip, wrist position, and finger movements influence spin type and effectiveness.
- *Feedback and correction*: by visualising the stationary coloured areas on the spinning ball in a global coordination system (GCS), coaches can give direct feedback on whether the desired spin is being achieved and the correct delivery technique in real-time. For instance, visualising the angular velocity vector in relation to pitch and yaw angles, coaches can provide targeted feedback to bowlers regarding their grip, arm action, and wrist movement. This can help improve their technique to produce more effective spins.

Furthermore, a more accessible form of coaching education and player feedback is available through plate carrée projections:

- *Less computationally intensive*: generating and rendering 2D projections is generally less resource-intensive than 3D visualisations, making it feasible to use simpler technology or software for analysis. A 3D visualisation would require a live 3D animation on a screen with active control to rotate the sphere to view the most appropriate perspective;
- *Easier to interpret*: plate carrée projections are simpler and often more intuitive to read than 3D representations. They provide a straightforward 2D view, making it easier to identify relationships between variables like pitch and yaw angles;
- *Direct correlation of angles*: the relationship between angles in a plate carrée projection is direct and linear, making it easier to visualise and analyse angular relationships without the complexities of perspective distortion present in 3D views;
- *Wide compatibility*: 2D projections like plate carrée can be more easily shared and viewed across various platforms and devices, including standard print media. For instance, plate carrée projections can be easily printed and shared in presentations or reports, making them more practical for educational and training contexts where discussions of specific techniques or performance analysis are needed.

In summary, the current smart ball research integrates both 3D representations and plate carrée projections in spin bowling biomechanics and coaching. This novel approach is essential due to the inherent complexity of spin bowling, which is often overlooked by conventional coaching methods. Traditional practices tend to underestimate the nuances of different hybrid deliveries, including variations in spin direction and swerve, as well as the intricacies of a gradient classification system that categorises these spins. As we enter the modern era of advanced coaching and biomechanics, it is crucial to embrace a comprehensive understanding of performance optimisation through the latest scientific advancements in motor control, skill acquisition, and learning. Coaches should no longer hide their reservations about scientific advances behind the slogan “keep things simple”. Utilising sophisticated tools like 3D representations and plate carrée projections allows coaches and players to effectively analyse and refine spin bowling techniques, ensuring that athletes can harness the full potential of their skills in a way that aligns with contemporary standards in sports science. This holistic approach not only enhances individual performance but also advances the overall strategy and effectiveness of spin bowling as a discipline.

## Figures and Tables

**Table 1:** Average and standard deviation of yaw and pitch angles (in degrees) of the angular velocity vector in the global coordinate system; intended deliveries were no-swerve; avg = average; act = actual; δ = distortion factor (δ-data are colour-coded for better readability); “*C* ” = consistency (standard deviation in degrees); corr. *C*_yaw_ = corrected consistency (=*C*_yaw_/δ); RI = righthander, LE = lefthander; FS = finger spin; WS = wrist spin; SS = sidespin, TS = topspin; TSS = top-sidespin; NS = no swerve; HS = half-swerve; if the actual delivery was different than the intended one, the actual delivery is displayed in bold italic font; perf. = performance; amat. = amateur bowler; 1st c. = first class bowler; elite = elite bowler; ω = angular velocity (spin rate, revolutions per second).

Bowler ID	Handedness	Perf. Level	Avg ω	Intended Delivery	Avg Yaw	Avg Pitch	Act. Yaw Delivery	Act. Pitch Delivery	*C*_yaw_	corr. *C*_yaw_	*C*_pitch_	δ
A1	R	amat.	19.8	FS-SS	70.0	25.6	***FS-TS***	***HS***	12.5	11.2	5.2	1.11
A2	R	amat.	18.1	FS-SS	82.0	46.4	***FS-TS***	***HS***	22.1	15.2	10.7	1.45
B1	R	amat.	30.2	FS-SS	–9.1	41.5	FS-SS	***HS***	13.6	10.2	18.9	1.33
B2	R	amat.	30.7	FS-SS	9.6	48.5	FS-SS	***HS***	29.2	19.3	19.7	1.51
C1	R	elite	37.0	WS-SS	76.1	40.5	***WS-TS***	***HS***	9.7	7.4	13.8	1.31
C2	R	elite	30.6	WS-SS	115.6	–11.5	***WS-TSS***	NS	11.8	11.5	15.6	1.02
D1	R	elite	29.0	WS-TSS	100.6	32.4	***WS-TS***	***HS***	17.0	14.3	12.7	1.18
D2	R	elite	32.0	WS-TSS	120.8	58.6	WS-TSS	***HS***	21.8	11.4	15.1	1.92
E1	L	ex-1st c.	30.0	WS-SS	–22.2	61.1	WS-SS	***HS***	33.3	16.2	7.5	2.06
E2	L	ex-1st c.	23.8	FS-TSS	138.7	–30.8	FS-TSS	***HS***	20.8	17.8	3.7	1.17
F	L	elite	25.7	FS-TSS	117.9	–8.0	FS-TSS	NS	14.5	14.3	9.4	1.01
G	L	1st c.	31.1	FS-TSS	109.6	35.3	***FS-TS***	***HS***	16.2	13.3	10.8	1.22

## Data Availability

The data presented in this study are available on request from the first author to any qualified researcher who has obtained ethics approval for secondary use of existing data through a consent waiver.
